# Supplementary material for: Perceptions of Sheep Farmers and District Veterinarians towards Sheep Disease Management in New South Wales, Australia
Source: Animals (Basel). 2024 Apr 22;14(8):1249. doi: 10.3390/ani14081249 (PMC11047500; doi:10.3390/ani14081249)
Supplement: Supplementary file 1 [file animals-14-01249-s001.zip › File S1 Farmers' sheep health questionnaire.pdf]

Copy of questionnaire

THE UNIVERSITY OF SYDNEY

Sydney School of Veterinary Science
Faculty of Science

425 Werombi Road, Camden, NSW 2570 AUSTRALIA

Associate Professor Om Dhungyel
Principal Research Fellow

Shute Building C01
Telephone +61 2 9351 1606
Facsimile +61 2 9351 1618
email om.dhungyel@sydney.edu.au

Farmers' sheep health and management survey questionnaire

Aims of the survey study

1. To understand common sheep health management practices and their impact at the farm level
2. To understand farmers' perception about sheep health and welfare and their impact on production
3. To understand the availability, accessibility and need for veterinary services and information.

Consent for Participation: I have read through the participation information and agree to the part of this questionnaire survey study. **Yes/No**

Property information and history:

Post code: ____________________________

Local Land Services (LLS) region ___________________________________

Producer/Manager (Please tick):

a. Gender: Male ☐ Female ☐ Other ☐

b. Age group: less than 30 years ☐ 31-50 years ☐ 51 years and over ☐

- **Property data:**

a. Total area (ha)_____________ Altitude: ____________________

b. Topography of paddocks (Please Tick)

Plains ☐ Undulating ☐ Steep hills ☐

c. Annual average rainfall (mm)_______________________________

d. Nearest BOM station ____

- **Pasture type and area (ha) (Please tick):**

Introduced perennial species ☐ ha:_________ Native species ☐ ha:______________

Fertilized native species ☐ ha:_________ Forage crops (winter) ☐ ha:____________

Forage crops (summer) ☐ ha:_________

- **Enterprise type (Please tick):**

Prime Lamb production ☐
Cropping (other than fodder) ☐ Mixed (Sheep and Cattle) ☐

Wool production: Fine (18 microns and less) ☐
Fine-Medium (18-19.5 microns) ☐
Medium (19.5 – 22 microns) ☐
Strong (22 microns upwards) ☐
Crossbred ☐

Non-wool (e.g. Dorper) ☐

- **Current sheep Breeds/numbers**

What is the total number of sheep currently on the property?__________________

	Current number of Stock
Lambing ewes	
Ewe hoggets	
Wethers	
Lambs	
Rams	

Do you have Merino sheep? Yes ☐ No ☐

Do you have other breeds of sheep? Yes ☐ No ☐
If Other breeds please specify:____

Type of flock Self Replacing ☐ Purchasing ☐

If purchasing what are the numbers per head (yearly average)?
Lambs:_______ Ewes______ Wethers____ Rams_____

- **Sheep management calendar (Please tick)**

	Jan	Feb	Mar	Apr	May	Jun	Jul	Aug	Sep	Oct	Nov	Dec
Lambing												
Shearing												
Crutching												
Dipping/Jetting												
Weaning												
Joining												
Drenching												

- **Neighbouring properties/share resources**

a. Number of neighbouring properties ____

b. Number of neighbours running sheep ____

c. Are any neighbouring boundaries on waterways? Yes ☐ No ☐

d. Rams shared with neighbours? Yes ☐ No ☐

i. **If yes,** how often? Frequently ☐ Sometimes ☐ Rarely ☐

e. Sheds/yards/roads shared with neighbours? Yes ☐ No ☐

f. Is there straying of sheep between properties? Yes ☐ No ☐

i. **If yes,** how often? Frequently ☐ Sometimes ☐ Rarely ☐

- **Property fencing**

What property fencing type do you have? Netting/Hinge joint/Plain wire/other (please circle)
If other, please specify_______________________________________

Boundary fencing type: Single ☐ Double fencing ☐

Boundary fence condition: Good ☐ Minor damage ☐ Poor ☐

Internal fence condition: Good ☐ Minor damage ☐ Poor ☐

- **Disease history**

a. Have any of the following sheep health concerns been present on your farm in the last 3 years? (Please Tick all that apply)

**Disease**	**Yes**	**No**
Internal Parasites		
External Parasites		
Ovine Johne's Disease		
Footrot		
Scabby Mouth		
Lameness		
Toxicity		
Abortion		
Clostridial Diseases (Includes: Blackleg, Botulism, Tetanus, Malignant Oedema, Red Water Disease, Black Disease and Pulpy Kidney)		
Other		

If answered other, please specify here ____________________________________

- **Sheep Mortality rate percentage (%) as a yearly average**

Lamb mortality	
Ewe mortality	
Wether mortality	
Ram mortality	

Disease control strategies:

- **Vaccination**

Do you use sheep vaccines? Yes ☐ No ☐

If yes, which vaccines? (choose all that apply)

6in1 ☐ 5in1 ☐ 3in1 ☐ Eryvac ☐ Scabigard ☐ Gudair ☐ Other ☐

If other vaccines, please specify:____

Are they given an annual booster? Yes ☐ No ☐

If you give the annual booster who do you give it to? Ewes ☐ Wethers ☐ Rams ☐

Following their initial vaccination, are lambs provided with a secondary vaccination? Yes ☐ No ☐

- **Parasite management**

Have you had drench resistance issues in your flock? Yes ☐ No ☐

Do you do regular parasite faecal egg counts? Yes ☐ No ☐

Do you do drench rotation? Yes ☐ No ☐

Do you follow Integrated parasite management? Yes ☐ No ☐

- **Biosecurity measures (Please tick/comment)**

a. Do you have a Farm Biosecurity Plan Yes ☐ No ☐

b. Do you inspect new stock for common diseases prior to purchase? Yes ☐ No ☐ Sometimes ☐

c. Do you quarantine new stock? Yes ☐ No ☐ Sometimes ☐

i. If yes or sometimes, for what period of time? __________________

j. Do you separate sheep with disease from the main flock? Yes ☐ No ☐ Sometimes ☐

i. If yes or sometimes, for what period of time?____________________

j. Prior to purchasing new stock, do you request for a Sheep Health Statement? Yes ☐ No ☐

k. Do you have quarantine areas/paddocks for new stock or sick animals on your farm? Yes ☐ No ☐

- **Economic Impact**

a. Please rate the following factors associated with common sheep diseases as a cause of **FINANCIAL** concern from 1-5 (1 = of greatest concern, 5 = of least concern)

Reason	Rating costs (1-5)
Veterinary	
Laboratory/diagnostic	
Vaccination	
Fencing	
Labour	

b. Who do you use for disease management? Contractors ☐ Farm labour ☐

- **Impact on animal welfare**

a. On your farm, do you think any of the following diseases are affecting animal welfare? (tick all that apply)

Lameness ☐ Internal Parasites ☐ External Parasites ☐ OJD ☐ Other ☐

If other diseases, please specify________________________________

b. How would you rate common diseases as a factor affecting the welfare of sheep on the property?

Low ☐ Moderate ☐ High ☐

c. How important to do you consider animal welfare to be in your enterprise?

Low ☐ Moderate ☐ High ☐

- **Access to services**

a. Where do you obtain the information for the control/prevention of disease? (Please tick/specify)

District vet Yes ☐ No ☐ Private vet Yes ☐ No ☐

Other__________________________________

b. Distance to veterinary services Far ☐ Too Far ☐ Near ☐

c. Do you feel there are adequate veterinary services in your district to manage disease occurrence and outbreak? Yes ☐ No ☐

d. How would you describe the ease of accessing veterinary assistance?

Hard ☐ Moderate ☐ Easy ☐

e. What is the availability of education programs/services for sheep management and disease control within your district?

Adequate ☐ Lacking ☐ Non-Adequate ☐ Could be improved ☐

f. When was the last time you contacted a vet for assistance/advice regarding the health of your sheep?

Less than 3 months ago ☐ 6 months ago ☐ 1 year ago ☐ >2 years ago ☐ Never ☐

g. Other than the DVs, what other sources of assistance of advice do you utilize? ______________________

- **Knowledge and information:**

a. What areas do you feel you would benefit having more knowledge around? (Tick all those that apply to you)

- ☐ Reproduction issues
- ☐ Common diseases
- ☐ Quarantine
- ☐ Biosecurity
- ☐ Parasite management
- ☐ Parasite lifecycles
- ☐ Which Vaccines to use
- ☐ Correct Vaccination Use
- ☐ Lamb mortality
- ☐ Trauma
- ☐ Nutrition advice
- ☐ Husbandry advice
- ☐ Sudden death of livestock
- ☐ Other __________________________
- ☐ None

b. How would you prefer to have information dispersed to you? (Tick all those that apply to you)

- ☐ In person
- ☐ Over the phone
- ☐ Famer Meetings/workshops
- ☐ At industry events
- ☐ Through pamphlets and other written media
- ☐ Through email
- ☐ At Town hubs (e.g sale yards, Pubs)
- ☐ Through collaboration with government bodies (e.g department of industries)
- ☐ Through collaboration with industry bodies and groups (e.g AWEC, MLA)
- ☐ Other_____________________

c. What do you consider to be the major problems facing sheep producers in your district in regards to sheep health? (Scale each option numerically from 1-10, with 1 = greatest concern, 10 = least concern)

**Sheep disease issue**	**Scale between 1-10**
Reproduction issues	
Disease outbreaks	
Parasitology	
Vaccination	
Lamb mortality	
Trauma	
Nutrition issues	
Husbandry issues	
Sudden death of livestock	
Other	

- **Producer opinion**
  a. Please mark the boxes which corresponds to your answer for each question regarding sheep disease management on your property.

Question/Comment	1 Strongly disagree	2 Disagree	3 Neither agree/disagree	4 Agree	5 Strongly agree
**General Statements**					
I consider disease a MINOR problem on the property					
I consider disease a MAJOR problem on the property					
I consider disease a significant cause of production losses					
I consider disease a significant cause of economic loss					
I consider disease management as a priority					
I am capable of dealing with most disease outbreaks					
In the event of most disease outbreaks I contact a vet					
It is important to inspect sheep for disease prior to purchase					
It is important to quarantine new stock prior to mix-up with the flock					

Additional Comments:____

____

____

____

Do you wish to receive a summary report of the overall findings of this survey study?
YES/NO.

If yes please write to the Chief Investigator Assoc. Prof. Om Dhungyel at om.dhungyel@sydney.edu.au

Thank you.
