# Supplementary material for: Perceptions of Sheep Farmers and District Veterinarians towards Sheep Disease Management in New South Wales, Australia
Source: Animals (Basel). 2024 Apr 22;14(8):1249. doi: 10.3390/ani14081249 (PMC11047500; doi:10.3390/ani14081249)
Supplement: Supplementary file 1 [file animals-14-01249-s001.zip › File S2 District Veterinarian questionnaire.pdf]

THE UNIVERSITY OF SYDNEY

Sydney School of Veterinary Science
Faculty of Science

425 Werombi Road, Camden, NSW 2570 AUSTRALIA

Associate Professor Om Dhungyel Principal Research Fellow

Shute Building C01
Telephone +61 2 9351 1606
Facsimile +61 2 9351 1618 email
om.dhungyel@sydney.edu.au

District Veterinarian sheep health survey questionnaire

Consent for Participation: I have read through the participation information and agree to the part of this questionnaire survey study. **Yes/No**

Aims of the questionnaire

1. Determine what form of information is most likely dispersed to producers
2. Identify what the major problems for producers are regarding sheep health
3. Identify the diseases that are the most common and difficult to control within districts
4. Determine what knowledge producers would benefit most from regarding disease management

District Veterinarian/Biosecurity Officer Information:

Local Land Services (LLS) region: ___________________________

1. What are the most common reasons you are called out to properties?
(Rank Numerically from 1-10, with 1 = most common and 10 = least common)

- ☐ Reproduction issues
- ☐ Disease outbreaks
- ☐ Parasitology
- ☐ Vaccination
- ☐ Lamb mortality
- ☐ Trauma
- ☐ Nutrition advice
- ☐ Husbandry advice
- ☐ Sudden death of livestock
- ☐ Other ___________________________________

2. **What areas do you feel farmers would benefit from having more knowledge around? (**Tick all that apply)
   - ☐ Reproduction issues
   - ☐ Common diseases
   - ☐ Quarantine and on farm disease management
   - ☐ Biosecurity
   - ☐ Parasite management
   - ☐ Parasite lifecycles
   - ☐ Which vaccines to use
   - ☐ Correct vaccination use
   - ☐ Lamb mortality
   - ☐ Trauma
   - ☐ Nutrition advice
   - ☐ Husbandry advice
   - ☐ Sudden death of livestock
   - ☐ Other____________________
   - ☐ None

3. **How are you most likely to disperse information to sheep producers?** (Rank Numerically from 1-10, with 1 = most likely and 10 = least likely)
   - ☐ In person
   - ☐ Over the phone
   - ☐ Famer Meetings/Workshops
   - ☐ At industry events
   - ☐ Through pamphlets and other written media
   - ☐ Through email
   - ☐ At town hubs (E.g sale yards, Pubs)
   - ☐ Through collaboration with government bodies (e.g department of industries)
   - ☐ Through collaboration with industry bodies and groups (E.g AWEC, MLA)
   - ☐ Other ________________________________

4. What do you consider to be the major problems facing sheep producers in your District in regards to sheep health?
(Rank numerically from 1-10, with 1 = greatest concern and 10 = least concern)

   - ☐ Reproduction issues
   - ☐ Disease outbreaks
   - ☐ Parasitology
   - ☐ Vaccination
   - ☐ Lamb mortality
   - ☐ Trauma
   - ☐ Nutrition issues
   - ☐ Husbandry issues
   - ☐ Sudden death of livestock
   - ☐ Other ________________________________

5. **In terms of endemic sheep diseases, which are the most common and difficult to control diseases in your district**
   (Circle each on a scale of 1-5, with 1 being severely concerning and 5 being of no concern)

Ovine Johne's Disease	1	2	3	4	5
Internal Parasites	1	2	3	4	5
External Parasites	1	2	3	4	5
Footrot	1	2	3	4	5
Clostridial Diseases	1	2	3	4	5
Other ___________________	1	2	3	4	5
